# The *Chlamydia pneumoniae* Tarp Ortholog CPn0572 Stabilizes Host F-Actin by Displacement of Cofilin

**DOI:** 10.3389/fcimb.2017.00511

**Published:** 2017-12-12

**Authors:** Rafat Zrieq, Corinna Braun, Johannes H. Hegemann

**Affiliations:** ^1^Department of Clinical Laboratory Sciences, College of Applied Medical Sciences, University of Ha′ il, Ha′ il, Saudi Arabia; ^2^Funktionelle Genomforschung der Mikroorganismen, Heinrich-Heine Universität Düsseldorf, Düsseldorf, Germany

**Keywords:** chlamydia, actin cytoskeleton, TARP, effector proteins, CPn0572, microbe-host cell interaction

## Abstract

Pathogenic *Chlamydia* species force entry into human cells via specific adhesin-receptor interactions and subsequently secrete effector proteins into the host cytoplasm, which in turn modulate host-cell processes to promote infection. One such effector, the *C. trachomatis* Tarp factor, nucleates actin polymerization *in vitro*. Here we show that its *C. pneumoniae* ortholog, CPn0572, associates with actin patches upon bacterial invasion. GFP-CPn0572 ectopically expressed in yeast and human cells co-localizes with actin patches and distinctly aberrantly thickened and extended actin cables. A 59-aa DUF 1547 (DUF) domain, which overlaps with the minimal actin-binding and protein oligomerization fragment required for actin nucleation in other Tarp orthologs, is responsible for the aberrant actin phenotype in yeast. Interestingly, GFP-CPn0572 in human cells associated with and led to the formation of non-actin microfilaments. This phenotype is strongly enhanced in human cells expressing the GFP-tagged DUF deletion variant (GFP-ΔDUF). Finally ectopic CPn0572 expression in yeast and *in-vitro* actin filament binding assays, demonstrated that CPn0572 stabilizes pre-assembled F-actin by displacing and/or inhibiting binding of the actin-severing protein cofilin. Remarkably, the DUF domain suffices to displace cofilin from F actin. Thus, in addition to its actin-nucleating activities, the *C. pneumoniae* CPn0572 also stabilizes preformed host actin filaments.

## Introduction

*Chlamydiae* are Gram-negative intracellular pathogens that infect a variety of organisms and tissues, and are responsible for several serious respiratory, ocular and urogenital diseases (Schachter, 1999). All *Chlamydia* species have a biphasic developmental cycle, alternating between the infectious but metabolically inert elementary body (EB) and the non-infectious, metabolically active reticulate body (RB). RBs replicate within a parasitophorous vacuole, termed an inclusion (Schramm et al., 1996; Belland et al., 2004). Successful uptake of EBs is crucial for *Chlamydia* infection, but the underlying molecular mechanisms are not well-understood.

Generally, the ability of bacterial pathogens to enter host cells depends upon cross-talk between bacterial and host factors, beginning with direct engagement of receptors on the target cell by adhesins, and/or translocation of effector proteins into the host-cell cytosol. These processes usually result in a rearrangement of the host-cell cytoskeleton, which in turn promotes a reorganization of the host plasma membrane architecture that facilitates bacterial uptake (Pizarro-Cerdá and Cossart, 2006). Initial attachment of the chlamydial EB is normally mediated by the interaction of the chlamydial surface protein OmcB with glycosaminoglycans (GAGs) on the host-cell surface, and is followed by more specific adhesin-receptor interactions (Hegemann and Moelleken, 2012). Thus, the *C. pneumoniae* adhesin/invasin Pmp21 binds directly to the human epidermal growth factor receptor (EGFR), activating signaling cascades that facilitate the uptake of *C. pneumoniae* EBs into their target cells (Mölleken et al., 2013). Moreover, the EB surface protein CPn0473 also mediates adhesion to human epithelial cells, and promotes EB uptake in a lipid-raft-dependent manner (Fechtner et al., 2016).

The *C. trachomatis* protein Tarp (translocated actin-recruiting phosphoprotein) is an early virulence effector protein implicated in host-cell invasion (Clifton et al., 2004; Lane et al., 2008; Jewett et al., 2010; Parrett et al., 2016). Tarp, which is assumed to be secreted by a Type-3 secretion system via Slc1 (SycE-like chaperone 1; CT043), is translocated into targeted cells within minutes of EB attachment, and associates with recruited actin at the site of bacterial attachment (Clifton et al., 2004; Brinkworth et al., 2011). This is accompanied by phosphorylation of several tyrosine residues near the N-terminus of Tarp by Src family tyrosine kinases and Ab1 kinase (Clifton et al., 2004; Jewett et al., 2008; Mehlitz et al., 2008). However, Tarp phosphorylation is not essential for chlamydia entry or actin recruitment. The protein most probably acts as a molecular scaffold to recruit host proteins that regulate actin dynamics and signaling events required for the early phase of chlamydial infection (Clifton et al., 2005; Jewett et al., 2008; Mehlitz et al., 2008; Thwaites et al., 2014).

Recruitment of actin to attached EBs early in the infection, in a pattern similar to that seen in *C. trachomatis*, has been observed for a number of chlamydial species (Clifton et al., 2005). However, although orthologs of the *C. trachomatis* Tarp gene are present in all sequenced *Chlamydia* species, they differ widely in amino acid sequence (displaying between 40 and 94% identity), domain structure and length (Clifton et al., 2005; Jewett et al., 2010; Jiwani et al., 2013), with the least conserved being the *C. pneumoniae* orthologs. For example, the *C. muridarum* and *C. pneumoniae* orthologs (but not the *C. caviae* ortholog) lack the tyrosine repeats (Clifton et al., 2005). In contrast, all Tarp orthologs harbor a protein oligomerization domain, and the actin-binding domains found in all examined chlamydial strains and species are able to nucleate actin polymerization *in vitro*. Interestingly, *C. pneumoniae* Tarp is the sole ortholog that has only a single actin-binding domain (Jewett et al., 2010). Recently, evidence was provided that the Tarp orthologs from serovars of *C. trachomatis* harbor two F-actin binding domains which seem to be absent from Tarp orthologs in other chlamydial species (Jiwani et al., 2013). Moreover, binding domains for the focal adhesion kinase (FAK) and for vinculin have been identified for Tarp proteins from various chlamydial species, but are not found in the *C. pneumoniae* ortholog (Thwaites et al., 2014, 2015).

To elucidate the functional consequences of these differences, we characterized CPn0572, the putative *C. pneumoniae* ortholog of Tarp. CPn0572 is secreted into the host-cell cytoplasm upon EB uptake and is associated with actin recruitment to the site of entry. Ectopically expressed CPn0572 stabilizes actin filaments (F-actin) both in yeast and in human HEK293T cells. Moreover, CPn0572 has the ability to generate or interact with other filamentous structures not associated with F-actin structures. Interestingly, detailed analysis revealed that CPn0572 blocks the disassembly of F-actin by displacing the F-actin destabilizing protein cofilin. Thus, in addition to the known function of Cpn0572 in nucleating F-actin, our findings provide evidence that the protein (like the prototypical Tarp from *C. trachomatis*) is a microbial F-actin-stabilizing protein. To our knowledge, this is the first bacterial effector protein that has been shown to directly modulate both actin nucleation/polymerization and depolymerization in host cells.

## Materials and methods

### Bacterial strains, eukaryotic strains and cell lines, and growth conditions

*Escherichia coli* (*E. coli*) XL-1 (Stratagene) was used for plasmid amplification and BL21 (Stratagene) for protein expression. Transformed *E. coli* strains were cultured in LB medium containing the appropriate antibiotics. *C. pneumoniae* GiD and *C. trachomatis* L2 (434) were propagated in HEp-2 cells (ATCC No.: CCL-23) and purified as described previously (Jantos et al., 1997; Wuppermann et al., 2008). The *Saccharomyces cerevisiae* strain CEN.PK2 (*MATa/MAT*α *ura3-52/ura3-52 trp1-289/trp1-289 leu2-3,112/leu2-3,112 his3-*Δ*1/his3-*Δ*1*) (EUROSCARF) was used for the homologous recombination cloning and the characterization of CPn0572 in yeast. The *aip1*Δ (*MATa his3*Δ*1 leu2*Δ*0 met15*Δ*0 ura3*Δ*0 YMR092c::kanMX4*) strain and the corresponding wt strain BY4741 (*MATa his3*Δ*1 leu2*Δ*0 met15*Δ*0 ura3*Δ*0*) (both from EUROSCARF) were used for cofilin (Cof1p) localization. Plasmid-bearing strains were grown on selective synthetic dextrose (SD) medium supplemented with 2% (wt/vol) glucose, raffinose or galactose (Sherman, 1991). HEK293T cells (ATCC No.: CRL-11268) were routinely cultured in IMDM medium (Invitrogen) supplemented with 10% (vol/vol) fetal calf serum (FCS; Invitrogen).

### Comparison of protein sequences

The following Cpn0572 and Tarp protein were used (accession numbers are included):

*C. pneumoniae* Cpn0572 CWL029 NP_224768.1
*C. trachomatis* Tarp LGV-434 AAT47185.1
*C. muridarum* TC0741 AAF39550
*C. caviae* CCA00170 AAP04921

Levels of similarity and identity between Cpn0572 and Tarp were calculated by EMBOSS http://emboss.bioinformatics.nl/cgi-bin/emboss/needle. Multiple sequence alignments of DUF domains from CPn0572 and its chlamydial orthologs were performed using MultAlin: http://bioinfo.genotoul.fr/multalin/multalin.html. The prediction of α-helices in DUF was carried out with the helical wheel projection prediction program (http://rzlab.ucr.edu/scripts/wheel/wheel.cgi).

### DNA manipulations and plasmid construction

Plasmids were constructed either by homologous recombination in *S. cerevisiae* or by ligation as indicated. In general, the plasmids used to generate the plasmids required for this study are listed in Table S1. The bacterial DNA sequences were amplified from genomic DNA or pre-existing plasmids using the oligonucleotides listed in Table S2. For expression of CPn0572_6His_ in *E. coli*, the pAC2 vector was cut at *Bgl*II and *Eco*RI sites and the coding sequence of *cpn0572* was integrated by homologous recombination in yeast. For expression of CPn0572 or its variants in mammalian cells, amplified DNA fragments were cloned into the pBYE vector (a modified pcDNA3.1/NT-GFP, see Table S1) at the *Acc*65I site. For yeast growth tests, the DNA coding sequences of *cpn0572* and its derivatives were cloned into the p426MET25 vector at the *Sma*I site (Mumberg et al., 1994). For localization studies in yeast *cpn0572* and its derivatives were also cloned into the pUG34 vector at the *Sma*I site via homologous recombination in yeast. Only the DNA fragment encoding GFP-C-terminal-ΔDUF was cloned into pUG34 by ligation. To do so, the DNA fragment was isolated from a p426MET25-GFP-C-terminal-ΔDUF vector by cleavage with *Spe*I and *Xho*I and cloned into the same sites in pUG34. To express GST fusion proteins in *E. coli, cpn0572* or the coding sequence for DUF or C-terminal-ΔDUF was amplified and integrated into the pKM36 vector at the *Sma*I site by homologous recombination. For expression of CPn0572 in the *aip1*Δ mutant strain and in wild type BY4741, the coding region of *cpn0572_6His_* was isolated from the p426MET25-cpn0572 vector using *Spe*I and *Xho*I, and integrated at the corresponding sites in p423GAL1 by ligation.

### Host-cell transfections and infection with *C. pneumoniae*

HEp-2 or HEK293T cells grown overnight on coverslips were transfected with 0.5–1 μg of the desired DNA plasmid using Turbofect (Thermo Scientific) as recommended by the manufacturer. Transfected cells were incubated in a CO_2_ incubator at 37°C for the indicated time periods (16 to 24 h). Human HEp-2 cells expressing mCherry-actin were infected with *C. pneumoniae* by adding purified EBs (suspended in cold DMEM medium) to attached HEp-2 cells, followed by centrifugation at 2,800 rpm, 4°C for 20 min. The medium was replaced by pre-warmed fresh medium and the cells were shifted to 37°C for the time periods indicated in the Figure legends. Samples were then prepared for microscopy as described below.

### Fluorescence microscopy and western blots

Yeast F-actin was stained with 6.6 μM rhodamine-phalloidin (Molecular Probes). To visualize cofilin and CPn0572_6His_, cells were fixed and indirectly immunostained as described previously (Pringle et al., 1991), using polyclonal chicken antibodies against cofilin (Okada et al., 2006) and a monoclonal mouse anti-His antibody (Novagen).

For indirect immunostaining infected or transfected human cells growing on glass coverslips were washed with 1x PBS, fixed in 3.7% (wt/vol) paraformaldehyde and permeabilized with 0.1% Triton X-100 (vol/vol). Permeabilized infected cells were then incubated with antibodies against *C. pneumoniae* MOMP (anti-MOMP antibody was kindly provided by G. Zhong, University of Texas Health Science Center, San Antonio, USA) to identify EBs and CPn0572 (anti-CPn0572 antibody was produced against the recombinant central domain of CPn0572 (aa S343 to A535) in rabbits by Eurogentec SA, Seraing, Belgium), and subsequently with labeled secondary antibodies. Staining of actin in human cells with rhodamine-phalloidin was performed as recommended by the manufacturer (Molecular Probes). Microscopy was carried out using either a spinning-disk confocal instrument or an Axiovert 200 microscope (Carl Zeiss).

For detection of GFP-tagged proteins from yeast on Western blots, a rabbit anti-GFP antibody (Molecular Probes) was employed, and for detection of proteins from human cells a mouse anti-GFP antibody (Thermo Scientific) was used.

### Disruption of the actin cytoskeleton

Yeast cells in mid-log phase were exposed to either Latrunculin-A (Lat-A) diluted in DMSO or to the solvent DMSO alone (final concentration 2.5 μM). At various times after addition of the agent, cells were removed, fixed immediately, and stained with rhodamine-phalloidin. The actin cytoskeleton of transfected HEK293T cells was disrupted by adding cytochalasin D (CD; Sigma) to the medium (final concentration 10 μM) for 30 min at 37°C.

### F-actin/cofilin co-sedimentation assay

F-actin was assembled *in vitro* as recommended by the supplier (Cytoskeleton Inc.). Equimolar amounts (2 μM) of recombinant human cofilin (Cytoskeleton) and pre-assembled F-actin were then incubated at pH 6.8 in the presence or absence of recombinant GST-CPn0572 (2 μM). The assay was then performed as described by the manufacturer.

## Results

### CPn0572 is secreted into HEp-2 cells early during infection by *C. pneumoniae* and associates with F-actin

**Figure 1:**
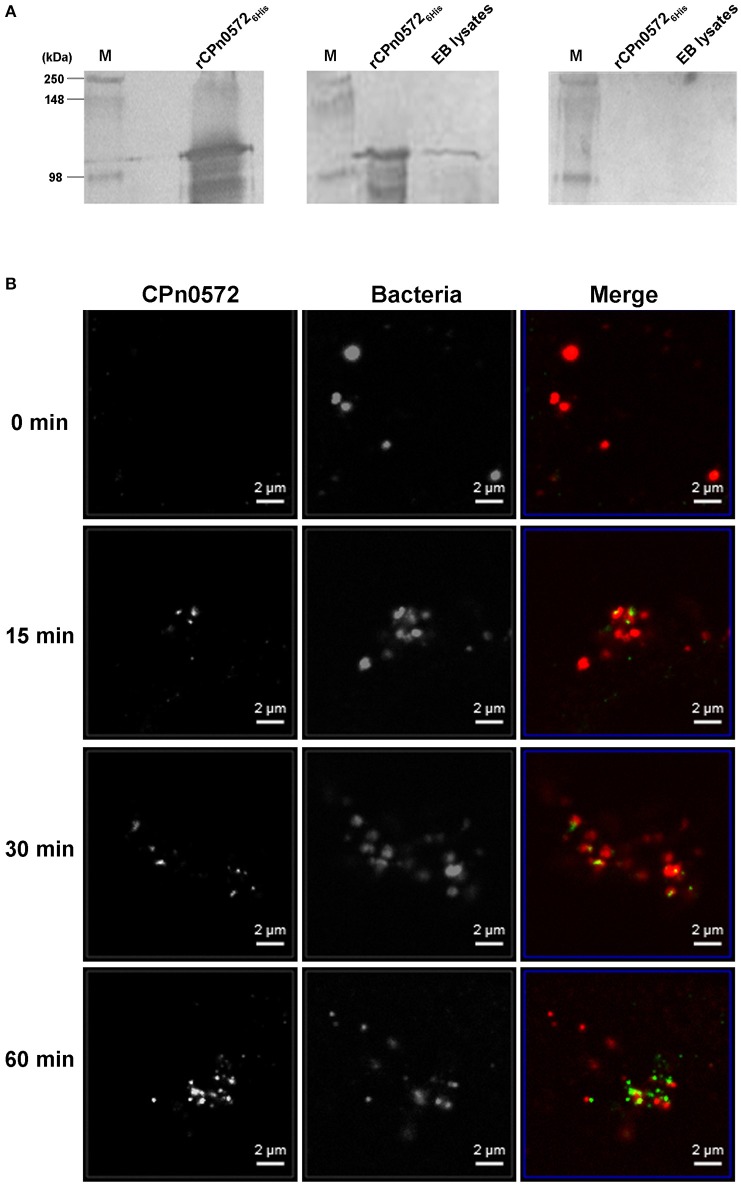
*C. pneumoniae* EBs express CPn0572 and secreted CPn0572 associates with F-actin. **(A)** CPn0572 is present in EBs. rCPn0572_6His_ protein and the endogenous protein expressed in *C. pneumoniae* EBs (EB lysates) were detected on immunoblots probed with a monoclonal anti-His antibody (left panel) and a polyclonal rabbit anti-CPn0572 antibody (middle panel), respectively. The blot shown on the right was probed with the corresponding preimmune serum. Full-scale images of the original blots are presented in Figure S1. **(B)** CPn0572 secretion increases over the first 60 min p.i. Secretion of CPn0272 was investigated at 0, 15, 30, and 60 min after HEp-2 cells had been infected with purified *C. pneumoniae* EBs (MOI = 50). EBs and CPn0572 were detected using anti-MOMP and anti-CPn0572 antibodies, respectively.

CPn0572 is currently annotated as a hypothetical *C. pneumoniae* protein. When we used a polyclonal antibody raised against the central region of the predicted ORF (CPn0572^S343−A535^) to probe western blots bearing protein extracts obtained from purified *C. pneumoniae* EBs, the antiserum (but not the corresponding preimmune serum) recognized a band of 110–115 kDa, similar in size to the recombinant full-length protein produced in *E. coli* (rCPn0572_6His_), indicating that CPn0572 is present in infectious EBs (Figure 1A and Figure S1).

**Figure 2:**
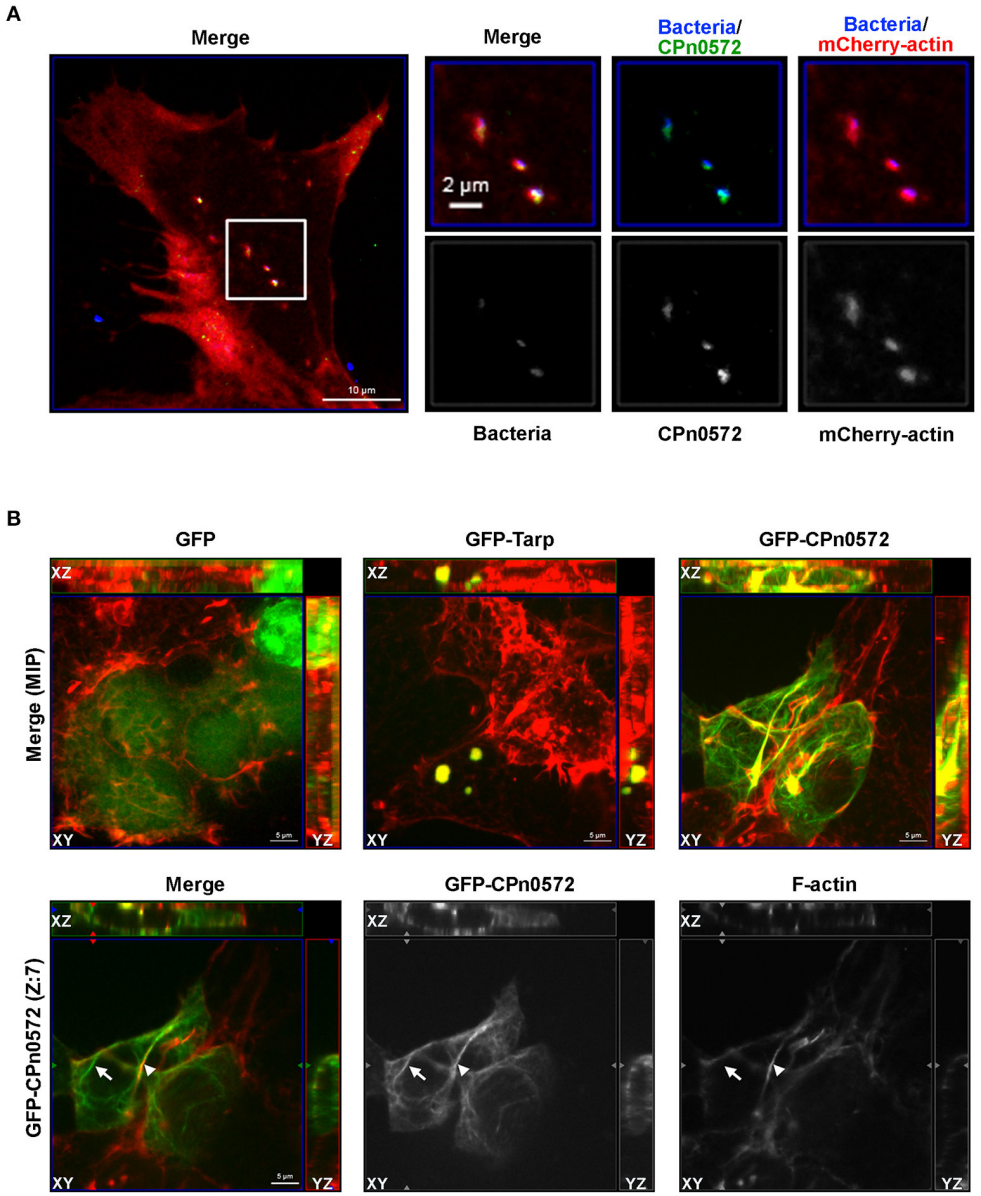
CPn0572 is associated with the host actin cytoskeleton in infected and transfected human cells. **(A)** Secreted CPn0572 colocalizes with host actin early in infection. HEp-2 cells expressing mCherry-actin (red) were infected with purified *C. pneumoniae* EBs (blue, MOI = 5). Secreted CPn0572 (green) is associated with actin recruitment to sites of EB entry at 30 min post-infection. The overall distributibbon of F-actin (red) is depicted in the merged image on the left; the boxed area is shown in greater detail on the right. **(B)** The effect of *C. pneumoniae* GFP-CPn0572 on the actin cytoskeleton differs from that of *C. trachomatis* GFP-Tarp. Upper row: Transfected HEK293T cells stained for GFP, GFP-CPn0572 or GFP-Tarp (green) and F-actin (red). The images shown are maximum intensity projections (MIP) of z-stacks. Lower row: A single section (Z: 7) from the z-stack of GFP-CPn0572 depicted in the upper row is shown. Note that not all GFP-CPn0572 filaments (arrows) coincide with actin fibers (arrowheads).

To determine whether the protein is translocated into the host-cell cytoplasm, infected HEp-2 cells were fixed at various time points after exposure to *C. pneumoniae*, and analyzed by immunofluorescence microscopy. At time 0, we observed no significant signal with anti-CPn0572 (Figure 1B). After 15 min, some bacteria were found to lie close to, or to partially overlap CPn0572 signals, as has previously been shown for the *C. trachomatis* Tarp and other secreted chlamydial proteins (Clifton et al., 2004; Hower et al., 2009). These results suggest that secretion of CPn0572 from the EBs had begun prior to this point. After 30 min and 60 min of infection, CPn0572 was detected in association with almost every EB, and the intensity of the signal increased over time (Figure 1B). To investigate whether EBs induce accumulation of actin at sites of entry, and to determine if CPn0572 is associated with actin, HEp-2 cells expressing mCherry-actin were infected with *C. pneumoniae* EBs. Adherent EBs were found to lie close by or immediately adjacent to CPn0572 signals and actin patches as early as 15 min p.i. (Figure 2A). These results indicate that CPn0572 is secreted from EBs into HEp-2 cells and is found in the vicinity of patches of F-actin accumulation at sites of EB entry.

### Tarp and CPn0572 show different patterns of localization and association with F-actin in transfected HEK293T cells

CPn0572 and its *C. trachomatis* ortholog Tarp show 22.9% sequence identity and 32.5% similarity; moreover, Tarp harbors an N-terminal extension of 276 amino acids (aa), while CPn0572 carries a unique 16-aa C-terminal extension. Interestingly, all Tarp orthologs harbor at least one copy of DUF, which overlaps with the minimal actin-binding and protein oligomerization region required for actin nucleation (Jewett et al., 2010). To determine whether CPn0572 expression in human cells results in an actin phenotype similar to that seen for the expression of *C. trachomatis* Tarp (Clifton et al., 2004; Jiwani et al., 2013), we expressed GFP, GFP-Tarp and GFP-CPn0572 in human HEK293T cells. In each cell expressing GFP-Tarp, the fusion protein was found exclusively in one or more discrete actin-containing structures (Figure 2B), similar to what has been described for HeLa cells expressing GFP-Tarp (Clifton et al., 2004; Jiwani et al., 2013). Interestingly, in all cells transfected with GFP-CPn0572, the fusion protein was detected not only in such actin-containing aggregates, but also in distinctly filamentous structures emanating from them (Figure 2B, merge, upper panel). Moreover, in more than 80% of the transfected cells, not all GFP-CPn0572-positive filaments were coincident with phalloidin-positive signals (Figure 2B; lower panel; compare the Cpn0572-positive fibers marked by an arrow and an arrowhead, respectively, and note the pair of Cpn0572-labeled non-actin filaments seen above the latter). As expected, GFP-CPn0572 expressed in human epithelial HEp-2 cells associated with actin aggregates and actin filaments but also formed fibers not associated with F-actin (Figure S2). These results suggest that CPn0572, like Tarp, associates with and alters the organization of the actin cytoskeleton independently of other chlamydial effectors. However, CPn0572 differs from Tarp in that it can also form fibers that apparently do not contain F-actin.

### CPn0572 inhibits yeast growth and DUF mediates disruption of F-actin

**Figure 3:**
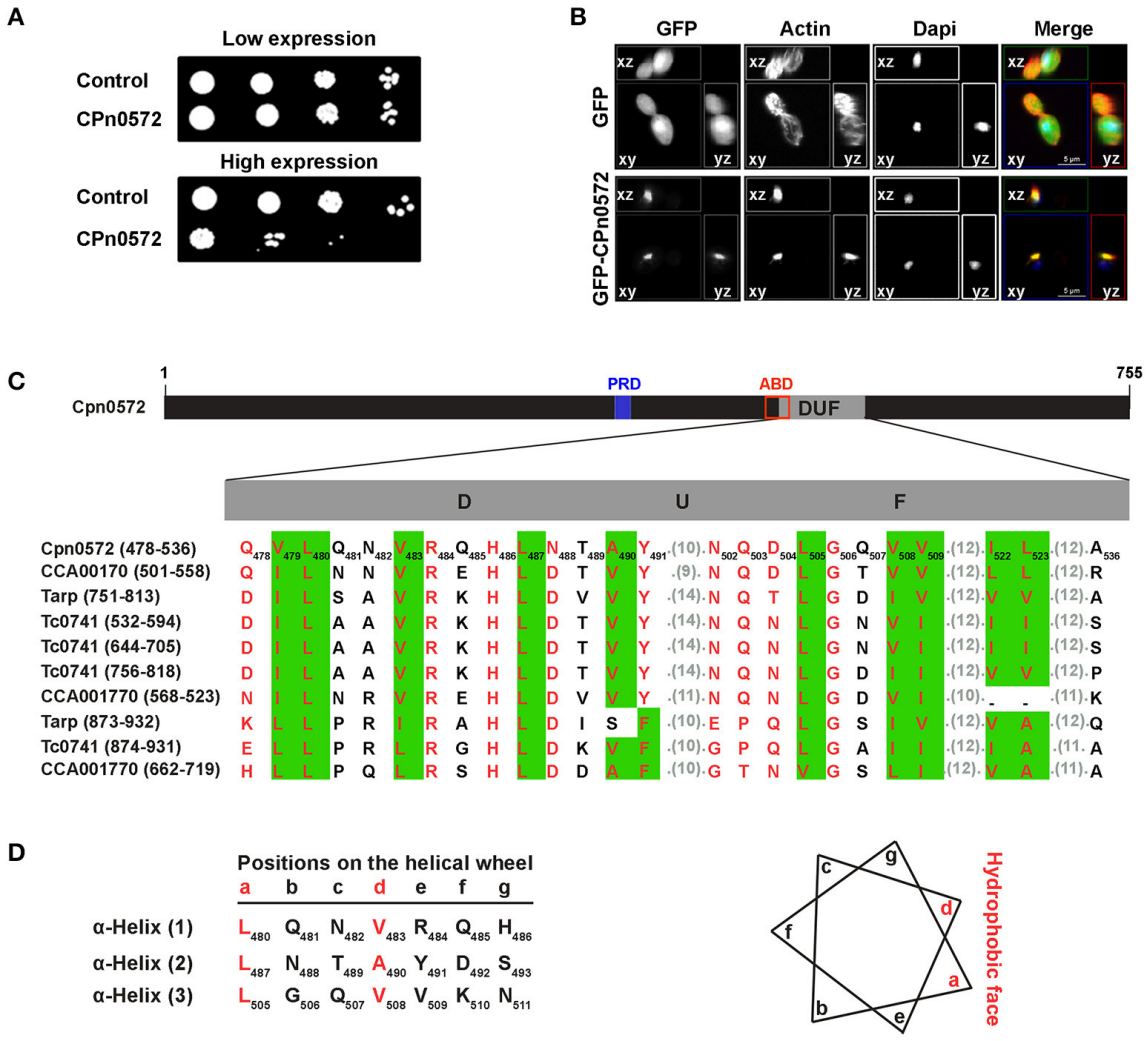
Expression of CPn0572 in *S. cerevisiae* inhibits growth and disrupts the actin cytoskeleton. **(A)** Serial-dilution patch tests of cells bearing either the empty plasmid (control), the repressed (low expression) or the induced (high expression) CPn0572 plasmid. **(B)** Cells expressing GFP (green, upper panel) or GFP-CPn0572 (green, lower panel) were stained for F-actin (red) and nuclei (blue). Data are shown as MIP images. **(C)** Schematic representation of CPn0572. The proline-rich domain (PRD, blue box) associated with the oligomerization capacity of Tarp and the potential actin-binding domain (ABD, red box; identified by Jewett et al., 2006, 2010) and the DUF domain are indicated. The conserved hydrophobic amino acids in DUF are predicted to form three α-helices. Multiple sequence alignment of DUF domains from CPn0572 and its chlamydial orthologs (*C. pneumoniae* Cpn0572, *C. trachomatis* Tarp, *C. muridarum* TC0741 and *C. caviae* CCA00170) as shown by MultAlin. The first and last amino acids of each DUF are indicated on the left. The number of each amino acid residue within the CPn0572 sequence is shown by the subscripts. Conserved amino acids are boxed in green. Hydrophobic amino acids are shown in red. Aligned residues are shown and the numbers of intervening unaligned residues in each sequence are given in parentheses. **(D)** Schematic representation of the disposition of the residues organized into α-helices (as indicated by the lower case letters) as depicted by the helical wheel projection prediction program. The amino acids at positions a and d (in red) represent the hydrophobic face of the wheel as shown on the right.

Because many of the processes and proteins in host cells that are altered or otherwise subverted during bacterial infections are conserved among eukaryotes (Valdivia, 2004; Siggers and Lesser, 2008; Zrieq et al., 2015), we used the yeast *S. cerevisiae* as a model system to investigate the role of CPn0572 in modulating cytoskeletal organization in greater detail. First, we expressed CPn0572_6His_ from an inducible promoter, and studied its effects on growth rate using serial-dilution patch tests. CPn0572_6His_-expressing cells displayed a severe growth defect, implying that CPn0572 disrupts an essential process in yeast (Figure 3A). We therefore examined the actin cytoskeleton in cells expressing GFP-CPn0572, and found the fusion protein in one large structure which colocalized with F-actin (Figure 3B). Moreover, a few of these aggregates were found to be associated with small numbers of short actin cables.

Collectively, these findings demonstrate that CPn0572 severely perturbs the yeast actin cytoskeleton by transforming actin structures into aggregates, as it does in human cells expressing GFP-CPn0572. Expression of the *C. trachomatis* Tarp in yeast causes a similar phenotype (Sisko et al., 2006). These data confirm the utility of yeast as a model for further studies of the role of CPn0572 in remodeling the actin cytoskeleton.

**Figure 4:**
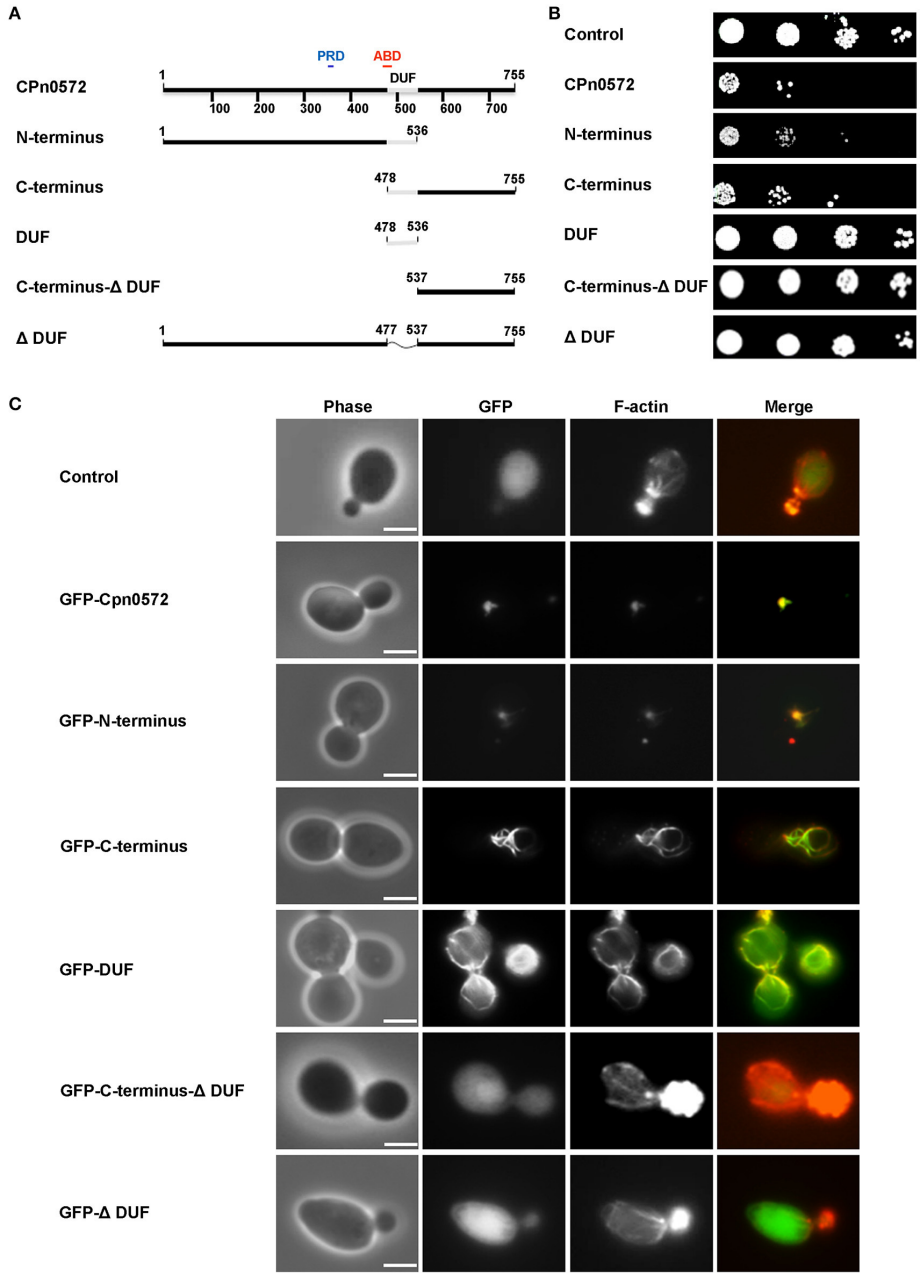
Effects of CPn0572 derivatives on growth rates and F-actin organization in yeast. **(A)** Schematic representation of the CPn0572 variants tested. The functional dissection of CPn0572 was based on the study of the DUF domain in combination with an N- or C-terminal segment of the protein (N-terminus and C-terminus, respectively), the C-terminus lacking DUF (C-terminus-DUF), the DUF domain alone (DUF) or the full-length protein deleted of DUF only (ΔDUF). The positions of these protein variants within the full-length protein are indicated by amino acid numbers. **(B)** Serial-dilution patch tests of yeast cells expressing full-length CPn0572 or the deletion variants indicated in **(A)**. **(C)** Actin organization in yeast cells expressing GFP fused to the full-length CPn0572 or its deletion variants indicated in **(A)**. Bar = 3 μm.

The DUF domain (59 amino acids) is the most striking element common to CPn0572 and its orthologs in other *Chlamydia* species (Figure 3C). It is predicted to consist of three α-helices (Figure 3D) and it forms part of the shortest fragment of CPn0572 (101 amino acids) found to pull down actin *in vitro*, while it overlaps partially with the potential actin-binding domain (ABD) (Jewett et al., 2010). Therefore, we suspected that DUF might be linked to the actin phenotype. Indeed, yeast cells expressing either an N-terminal (M^1^-A^536^) or C-terminal (Q^478^-K^755^) segment of CPn0572 including DUF displayed a growth phenotype like that seen with the full-length CPn0572 (Figure 4B), while deletion of the DUF domain alone (Q^478^-A^536^, ΔDUF, Figure 4A) restored normal growth of yeast cells expressing this variant (Figure 4B). In contrast, expression of the DUF domain (Figure 4A) on its own had no effect on growth (Figure 4B). Interestingly, deletion of DUF domain from the C-terminal fragment (C-terminal-ΔDUF, Figure 4A) restored normal yeast growth (Figure 4B; compare C-terminus vs. C-terminal-ΔDUF). Taken together, these results imply that DUF is essential, but not sufficient, for the severe growth phenotype.

We then examined the actin cytoskeleton in yeast cells expressing the different Cpn0572 variants. The GFP-N-terminal segment of the fusion protein colocalized with actin structures comparable to those induced by the full-length protein (Figure 4C). Interestingly, cells expressing the GFP-C-terminal segment showed a very different actin pattern, characterized by complete colocalization of the fusion protein with long, thick actin cables and little cytosolic GFP-C-terminus staining (Figure 4C). In contrast, cells expressing the GFP-C-terminal-ΔDUF segment showed typical actin cables and patches found in wild-type cells and a diffused distribution of the fusion protein comparable to that of cells expressing GFP alone, indicating that the lack of toxicity and actin remodeling is due to the absence of the DUF domain (Figure 4C). As expected, cells expressing the GFP-ΔDUF construct showed diffuse GFP staining in the cytosol, and their actin cytoskeleton was unaffected (Figure 4C). Finally, GFP-DUF itself was found to stain distinctive, unusually shaped and mostly cortically localized actin cables against a strong and diffuse background of the protein in the cytosol (Figure 4C). These data indicate that the DUF region of CPn0572 is crucial for the association of CPn0572 with F-actin (Figure 4C). The differences in the localization of the different GFP-CPn0572 segments are unlikely to be due to major alterations in protein expression levels (Figure S3A). Taken together, these observations strongly suggest that DUF is required for the association of CPn0572 with F-actin in yeast cells, while other elements further modulate the actin phenotype, which then impairs yeast cell growth.

**Figure 5:**
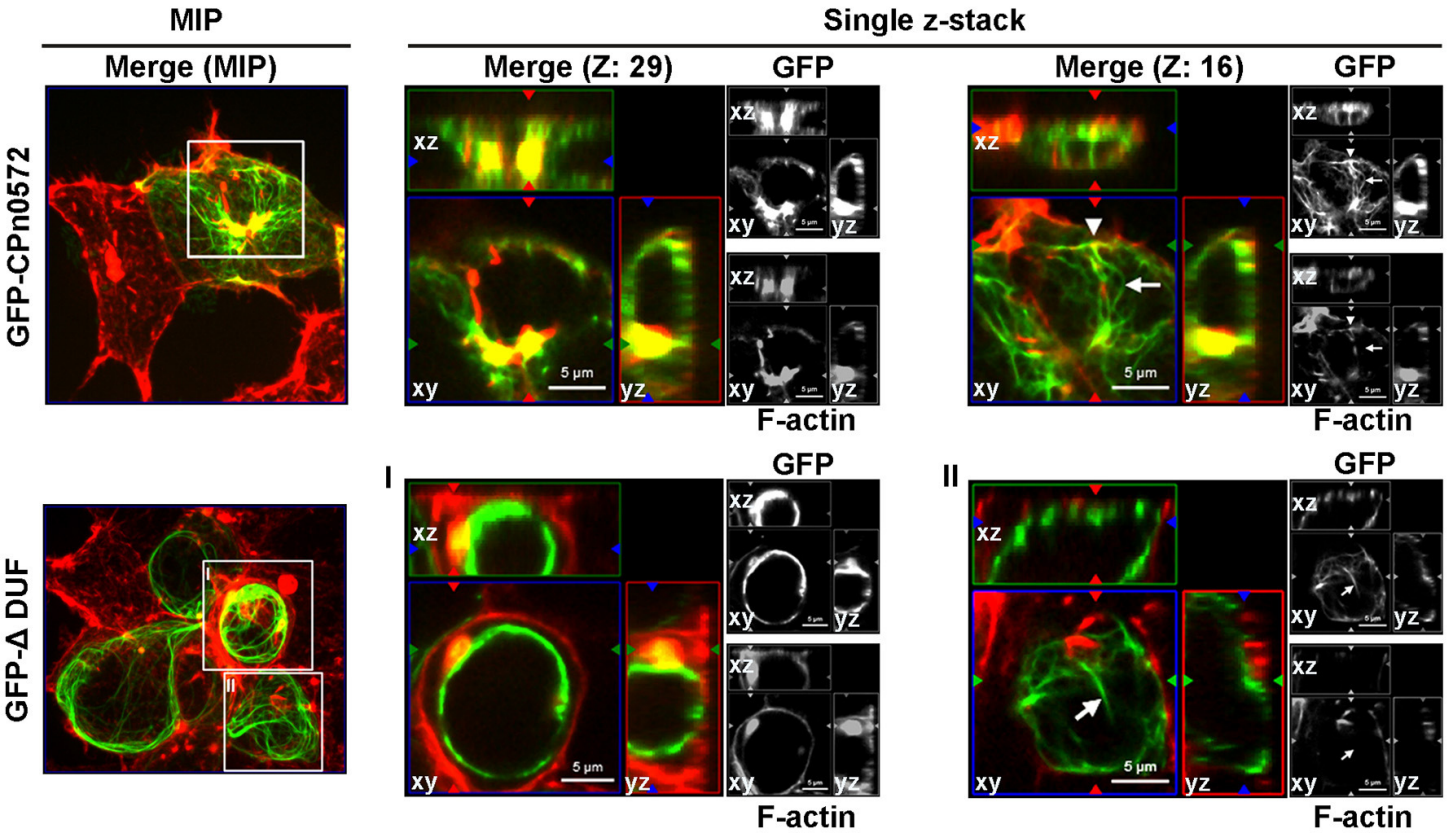
GFP-ΔDUF associates with both actin and non-actin filaments. The actin cytoskeleton (red) in HEK293T cells expressing GFP-CPn0572 (green, upper row) or GFP-ΔDUF (green, bottom row) is shown as MIP images (from stacks of 40 sections) **(left)** and selected single z-stacks **(right)**, as indicated. The enlarged images of single z-sections correspond to the boxes in the MIP images (one box from GFPCPn0572: sections Z: 29 and Z: 16; boxes I and II from GFP-ΔDUF). GFP-CPn0572 cables made up of F-actin are indicated by arrowheads, while GFP-CPn0572- or GFP-DUF-bound cables not containing F-actin fibers are marked by arrows.

Next we tested these findings in human cells. As shown above, GFP-CPn0572 was found to localize to complex actin structures, and all CPn0572-expressing cells showed the fusion protein filaments colocalizing with some, but not all actin fibers (Figure 5, upper panel, Z: 29; compare arrow and arrowhead in Z: 16). In contrast, in more than 80% of transfected cells, most GFP-ΔDUF did not colocalize with actin fibers (Figure 5, lower panels; see arrow in Z: 16), although this variant was occasionally associated with F-actin aggregates (Figure 5, lower panels, MIP and Z: 29). Strikingly, however, the vast majority of GFP-ΔDUF was found in the form of filamentous structures that did not contain actin (Figure 5, lower panel). The phenotypic differences seen for GFP-CPn0572 and GFP-ΔDUF are likely not due to differences in protein expression (Figure S3B). These results confirm that DUF is required for the association of CPn0572 with F-actin in human cells. Moreover, CPn0572 has a DUF-independent ability to generate or interact with other filamentous structures.

### CPn0572 stabilizes F-actin by inhibiting its depolymerization

**Figure 6:**
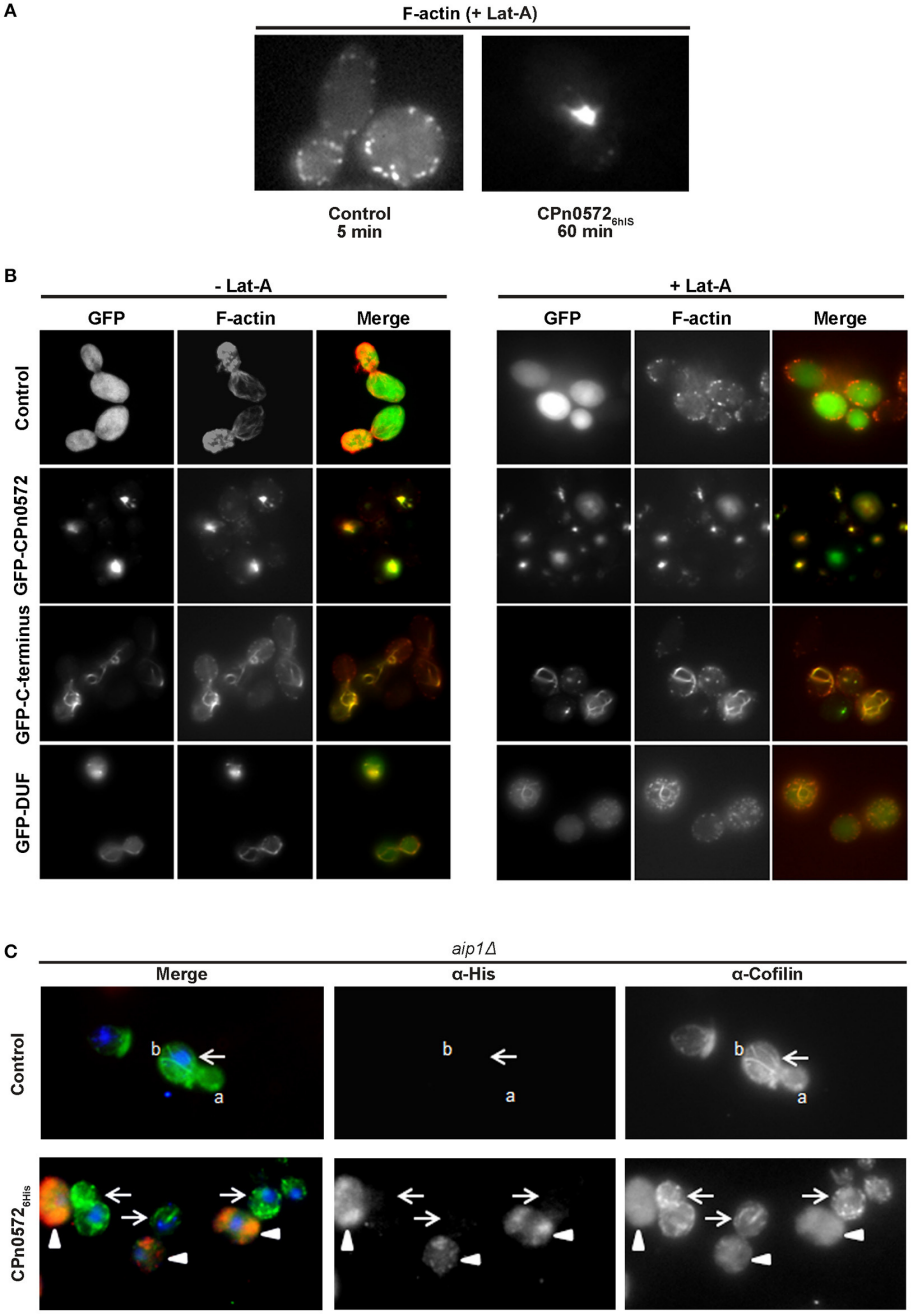
CPn0572 and its C-terminal segment stabilize F-actin and displace cofilin from F-actin in yeast *in vivo*. **(A)** GFP- (control) or GFP-CPn0572-expressing cells were treated with Lat-A (+ Lat-A) for the indicated times, and stained for F-actin. **(B)** Actin cytoskeleton patterns in yeast cells expressing full-length CPn0572 or its deletion variants before and after treatment with Lat-A. Yeast cells expressing GFP (control), GFP-CPn0572, GFP-C-terminus or GFP-DUF were first grown to mid-log phase in inducing medium, exposed to DMSO alone (– Lat-A) or Lat-A (final concentration 2.5 μM) in DMSO (+ Lat-A) for 1 h, then fixed and stained with rhodamine-phalloidin to visualize F-actin, and examined using an Axiovert 200 microscope (Carl Zeiss). **(C)** In *aip1*Δ yeast cells carrying the empty vector (control), cofilin (green) localizes to actin patches and cables (marked by a and b, respectively). In cells expressing CPn0572_6His_ (red), cofilin is diffusely distributed (arrowheads), while non-expressing cells retain distinct cofilin patches and cables (arrows). See also Figure S4.

Ectopic expression of CPn0572 in eukaryotic cells induces aggregation of F-actin into thick cables, which resemble analogous structures observed in yeast and human cells under conditions in which actin polymerization is favored over F-actin depolymerization (Ayscough, 2000; Lázaro-Diéguez et al., 2008). In part this can be explained by CPn0572's known ability to nucleate and promote the polymerization of F-actin *in vitro* (Jewett et al., 2010). However, the fact that in human cells CPn0572 exhibits continuous colocalization with distinct actin fibers suggested that CPn0572 might also stabilize F-actin against the action of actin-depolymerizing agents. To test this hypothesis, yeast cells expressing GFP alone or GFP-CPn0572 were exposed to Latrunculin A (Lat-A), which binds actin monomers, thus preventing their polymerization and effectively promoting depolymerization (Coué et al., 1987; Ayscough et al., 1997; Belmont and Drubin, 1998; Okada et al., 2006). Control cells expressing GFP alone were devoid of actin cables after treatment with Lat-A for 5 min, while some cortical actin patches were still visible after 1 h of exposure to Lat-A (Figure 6A). In contrast, the massive actin structures induced by GFP-CPn0572 were still detectable even after exposure to Lat-A for 1 h. Similarly, yeast cells expressing the GFP-C-terminus or GFP-DUF retained actin cables; however, in the presence of Lat-A, the GFP-C-terminus associated with more distinctively defined (compared to the GFP-DUF construct) cables and patches which were also phalloidin-positive (Figure 6B). Thus, CPn0572 stabilizes F-actin in yeast by inhibiting depolymerization *in vivo*.

### CPn0572 displaces cofilin from F-actin structures

ADF/cofilins are key regulatory proteins that bind to and sever F-actin *in vitro* and *in vivo* (Elam et al., 2013). We therefore tested the hypothesis that CPn0572 might alter F-actin dynamics by interfering with cofilin function. We determined the localization of cofilin in yeast by expressing CPn0572_6His_ in an *aip1*Δ strain, in which both actin patches and cables are enriched for cofilin, and cofilin can be visualized on all actin structures (Okada et al., 2006). In *aip1*Δ cells carrying the empty vector, cofilin was detected in structures interpreted to represent actin cables and patches, as previously documented (Figure 6C, upper panel). Remarkably, in CPn0572_6His_-expressing cells, this clear localization of cofilin was completely abolished, and only a diffuse cytosolic staining pattern was detectable (Figure 6C, lower panel; CPn0572_6His_-expressing cells marked by arrowheads), while in the same micrograph, yeast cells with little or no CPn0572_6His_ expression retained actin cables and patches (Figure 6C, lower panel; cells marked by arrows). Similarly, CPn0572_6His_ also altered cofilin localization in a wild-type yeast strain (Figure S4). These results strongly suggest that CPn0572 inhibits F-actin depolymerization in yeast by displacing cofilin from F-actin.

**Figure 7:**
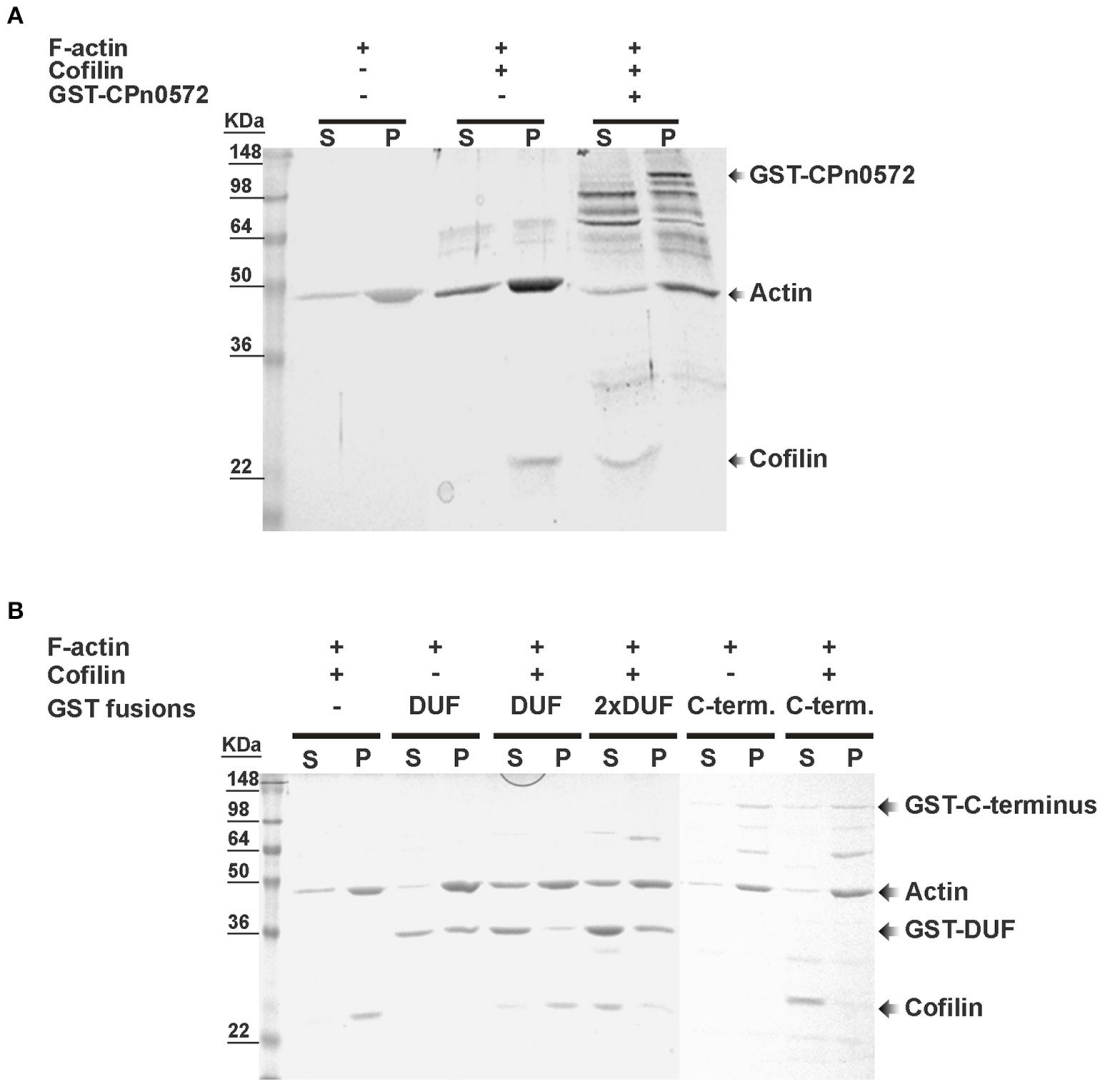
Displacement of mammalian cofilin from preassembled mammalian F-actin by GST–CPn0572 and GST-coupled domains of CPn0572 *in vitro*. **(A)** Binding of GST-CPn0572 and cofilin to pre-assembled F-actin was analyzed using a co-sedimentation assay. Supernatant (s) and pellet (p) fractions were analyzed by SDS-PAGE and stained with Coomassie blue. The presence (+) or absence (–) of each protein in the reaction mix is indicated. The corresponding protein band is marked. **(B)** The experiment was performed as described in **(A)**, using 2 μM GST, GST-DUF (DUF) or GST-C-terminus (C-term.) or 4 μM GST-DUF (2x DUF). Supernatant (s) and pellet (p) fractions were analyzed by SDS-PAGE and stained with Coomassie blue. The presence or absence of each protein is indicated. The corresponding protein band is marked. A full-scale image of the original blot is presented in Figure S5.

Next we used *in vitro* assays with recombinant proteins to test whether the displacement of cofilin from filamentous actin by CPn0572 was a direct or indirect effect of the latter. Pre-assembled mammalian F-actin was mixed with recombinant human cofilin in the absence or presence of an equivalent amount of GST-CPn0572, and the mixture was subjected to ultracentrifugation. When F-actin was mixed with cofilin alone, 100% of the cofilin was found in the pellet, as expected given its ability to bind to F-actin (Figure 7A). However, when recombinant GST-CPn0572 was present in the mixture, cofilin was now found exclusively in the supernatant, and the fusion protein was found in the pellet with F-actin. Thus, these results show that CPn0572 binds F-actin and competes with cofilin for, and/or displaces it from, F-actin. Further analysis revealed that the GST-C-terminus displaced about 90% of the cofilin into the supernatant. Finally, the GST-DUF domain was able to displace only about 20% of the cofilin into the supernatant and a 2-fold excess of DUF was necessary to bring about 80% of the cofilin into solution *in vitro* (Figure 7B). These results are consistent with our finding that DUF displays less colocalization with F-actin, and is a less potent stabilizer of F-actin in the presence of Lat-A. Thus, the ability of CPn0572 to prevent binding of cofilin to F-actin largely resides in its C-terminal segment.

**Figure 8:**
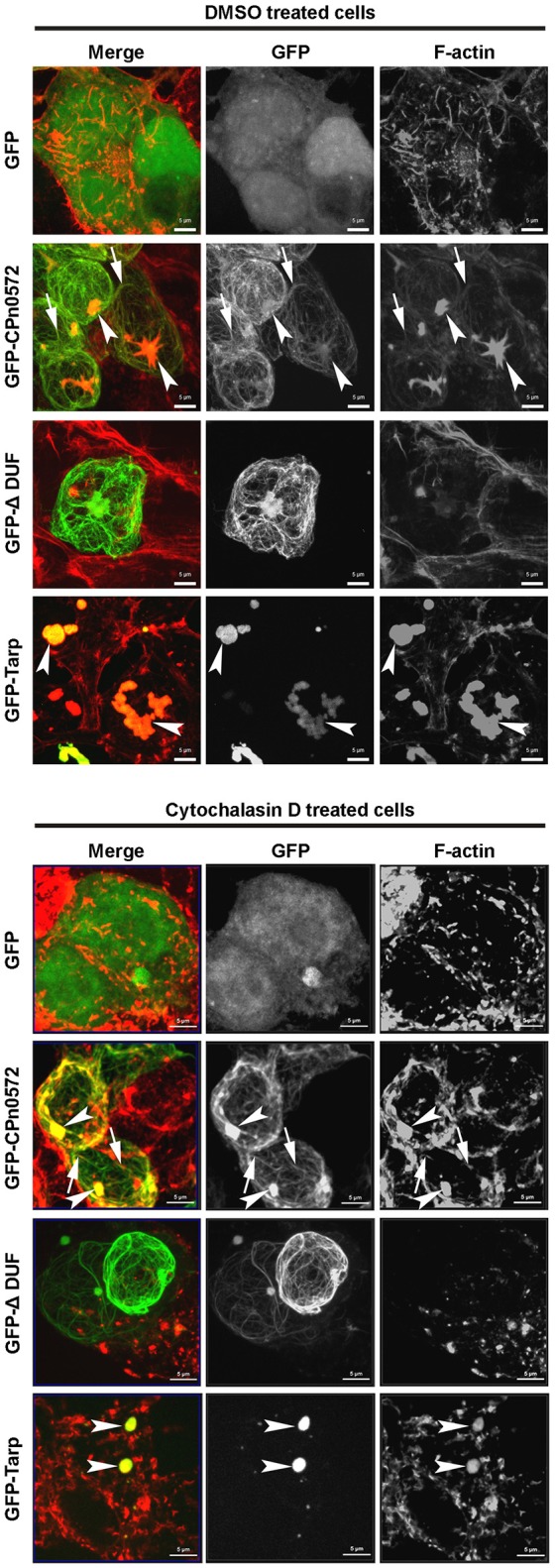
Both CPn0572 and Tarp stabilize the actin cytoskeleton in HEK293T cells. F-actin staining of GFP-, GFP-CPn0572-, GFP-CPn0572-ΔDUF- or GFP-Tarp-expressing HEK293T cells exposed to the solvent DMSO **(upper)** or after treatment with cytochalasin D **(lower)**. The actin cytoskeleton is shown in red and GFP signals in green. GFP-CPn0572 cables associated with F-actin are indicated by arrows, while GFP-CPn0572 and GFP-Tarp associated actin aggregates are marked by arrows. Data are shown as MIP images.

We also probed the ability of CPn0572 to stabilize F-actin in human cells. When we destabilized F-actin by incubating HEK293T cells with cytochalasin D (CD), which inhibits actin filament dynamics in cells via multiple mechanisms, GFP-expressing control cells showed depolymerized F-actin with some dispersed cortical actin remaining intact (Figure 8, upper and lower panels). In contrast, CD had little effect on actin structures and fibers in HEK293T cells expressing GFP-CPn0572 in comparison to untreated cells (Figure 8, upper and lower panels). Interestingly, the actin aggregates induced by the *C. trachomatis* Tarp were also resistant to CD (Figure 8). Most interestingly however, treatment of HEK293T cells expressing a GFP-ΔDUF fusion with CD resulted in the complete loss of F-actin structures. Moreover, no colocalization of the remaining cortical actin with GFP-ΔDUF was observed, supporting our contention that the DUF domain is crucial for stabilization of F-actin by CPn0572 (Figure 8). Overall, these data show that CPn0572 and Tarp stabilize F-actin against depolymerizing and severing factors present in human cells.

## Discussion

The actin cytoskeleton is regarded as an important target for reorganization during *Chlamydia* uptake. Previous studies indicated that the *C. trachomatis* Tarp protein binds G-actin and nucleates its polymerization *in vitro* (Clifton et al., 2004; Jewett et al., 2006). However, previous work had also suggested that the *C. pneumoniae* Tarp ortholog might be functionally distinct in some respects from other Tarp proteins (see Introduction). In this work, we show that the *C. pneumoniae* ortholog of Tarp, CPn0572 (previously classified as “hypothetical”), is expressed in EBs (Figure 1A) and secreted into HEp-2 cells early during infection (Figure 1B). During infection secretion of CPn0572 is detectable as early as 15 min after EB attachment. Thus, CPn0572 and Tarp are both translocated into the host cell, and are available at the site of bacteria entry. The fact that we could not observe CPn0572 signals at time point zero (Figure 1B) is consistent with the results obtained previously for Tarp (Clifton et al., 2004). Very likely, the antibody does not reach the bacterial cytoplasm during immunostaining.

After infection, secretion of CPn0572 continues for at least the first 60 min p.i. and the protein becomes associated with actin patches within 15 min after EB attachment. The *in vitro* data indicate that CPn0572 binds directly to F-actin (Figure 7A). This corroborates and extends biochemical data which demonstrated that CPn0572 is capable of binding G-actin and nucleating F-actin formation *in vitro* (Jewett et al., 2010). Thus, CPn0572 and Tarp share the same actin nucleation activity *in vitro* and show the same actin-recruiting activity early in infection *in vivo*; both functions are most probably essential for entry of the respective chlamydial species into their target cells (Clifton et al., 2004; Jewett et al., 2010).

Expression of CPn0572 in yeast has a very detrimental effect on growth, and converts the typical yeast actin cytoskeleton into a large aggregate very like that induced by the Tarp expressed by *C. trachomatis* serovar L2 (Sisko et al., 2006). The growth defect indeed results from the disruption of the actin cytoskeleton, since removal of the actin-binding domain DUF (aa 478 to aa 536) from CPn0572 restores both wild-type actin organization and growth rate. Conversely, CPn0572 binds actin *in vitro* via its DUF domain (Figure 7B). Moreover, it may well oligomerize via a proline-rich domain within its N-terminal segment (aa 349 to aa 371) as shown for the prototypic *C. trachomatis* Tarp (Jewett et al., 2006). Indeed, expression of a CPn0572 truncation mutant that lacks only the segment C-terminal to the DUF domain in yeast induces an actin phenotype almost identical to that induced by the full-length protein (Figure 4B). In contrast, the CPn0572 C-terminal segment including DUF does not form actin aggregates, but colocalizes with extra-long and thickened actin cables in yeast and also reduces growth rate, suggesting that the C-terminus also harbors actin-modulating capacities. Recently, two F-actin-binding domains (FAB1 and FAB2) have been described in the C-terminus of the *C. trachomatis* Tarp, the latter overlapping with a vinculin-binding site (VBS3) in the *C. caviae* GPIC Tarp (Jiwani et al., 2013; Thwaites et al., 2015). It is worth noting that FAB and VBS-like sequences have been found in Tarp orthologs from various chlamydial species, but not in CPn0572, which once again points to functional differences between Tarp proteins and CPn0572 (Jiwani et al., 2013; Thwaites et al., 2015). Interestingly, when the CPn0572 DUF domain alone (aa 478 to aa 536) is fused to GFP, the fusion protein colocalizes with actin patches and distinctly thickened and extended actin cables, indicating that the 59-aa DUF domain is sufficient for binding and bundling of F-actin. This is compatible with the *in vitro* actin-binding activity previously reported for a longer actin-binding segment (aa 440 to aa 540) of the protein (Jewett et al., 2010). In fact, the action of the CPn0572 DUF domain on F-actin in yeast is reminiscent of the actin phenotype induced by the *Salmonella* F-actin stabilizing protein SipA (Lesser and Miller, 2001).

As in yeast, ectopic expression of either CPn0572 or *C. trachomatis* Tarp in human cells induces an actin aggregation phenotype; however, only the former shows a continuous colocalization with distinct actin fibers (Figure 2B). Not only that, Cpn0572 was often seen to form filamentous structures that are apparently devoid of actin (e.g., Figure 5). The nature of these CPn0572-positive fibers and their relationship to other cellular structures are unknown at present.

Interestingly, the CPn0572-induced actin structures found upon expression of the protein in yeast and human cells are resistant to the action of the actin-depolymerizing drugs Lat-A and CD (Figures 6, 8), which suggests that CPn0572 might stabilize F-actin against depolymerization. This phenotype is comparable to those described previously for yeast cells exposed to the actin-nucleating and stabilizing toxin jasplakinolide (Jpk), which inhibits actin depolymerization both *in vitro* and *in vivo* (Bubb et al., 1994; Ayscough, 2000; Vallotton et al., 2004; Lázaro-Diéguez et al., 2008). Jpk-induced F-actin fibers are resistant to the actin-depolymerizing drug Lat-A in yeast and to Lat-B in human cells (Ayscough, 2000; Lázaro-Diéguez et al., 2008).

Cofilin plays a central role in promoting actin turnover by severing/depolymerizing F-actin in all eukaryotic cells, and we therefore tested its potential role in CPn0572-induced actin stabilization (Elam et al., 2013). Indeed, upon CPn0572 expression in yeast, cofilin is excluded from the CPn0572-induced actin aggregates *in vivo* (Figure 6C). Thus, CPn0572 likely stabilizes F-actin via a mechanism that involves displacement of cofilin from F-actin. That Cpn0572 is indeed capable of displacing cofilin was confirmed biochemically using purified mammalian F-actin, recombinant human cofilin and recombinant CPn0572 *in vitro* (Figure 7A). Remarkably, the DUF domain suffices to displace cofilin from pre-assembled F-actin (Figure 7B). Interestingly, α-helix 3 of cofilin binds in the hydrophobic cleft located between subdomains 1 and 3 of actin (reviewed in Dominguez, 2004), and the α-helical structure found in DUF is compatible with the hypothesis that CPn0572 and cofilin compete for binding to the same cleft in actin. The ability of CPn0572 and Tarp to render actin aggregates and filaments resistant to CD in transfected HEK293T cells provides evidence that both proteins can stabilize F-actin not only against the action of cofilin but against other, perhaps even all, actin-depolymerizing and severing factors.

F-actin recruitment and stabilization by CPn0572 is very likely integrated with other entry-related processes. Adhesion of *C. pneumoniae* EBs to human cells occurs via the Pmp proteins, which bind and activate the EGF receptor, and signaling by the EGFR via Erk1/2 and the LIM kinase may lead to phosphorylation of cofilin, thereby inhibiting its actin-binding, filament-severing and depolymerizing activities, all of which may contribute to cytoskeletal changes during endocytosis of EBs.

ADF/cofilin is the central F-actin severing factor in human cells, and thus it is not surprising that modulation of its activity is also exploited for host-cell invasion by other intracellular bacteria and by viruses (Zheng et al., 2016). For example, *Listeria* activates the LIM kinase, which disables cofilin via phosphorylation by an unknown factor, thus preventing excessive depolymerization of the F-actin network upon entry of *Listeria* (Bierne et al., 2001). In *Salmonella*, SipA stabilizes F-actin by inhibiting the severing and depolymerizing activities of ADF/cofilin and gelsolin (McGhie et al., 2004), while a second effector, SipC, induces actin polymerization (Hayward and Koronakis, 1999). SipA appears to augment the activity of SipC (McGhie et al., 2004). Thus, F-actin nucleation and F-actin stabilization by displacement of cofilin are induced by two different *Salmonella* effectors, while in *Chlamydiae* the two functions have been co-opted into one protein. Actin assembly requires a flux of G-actin, which is normally provided by active actin depolymerization elsewhere in the cell. Cofilin displacement by CPn0572 should therefore enhance depolymerization of F-actin structures not associated with CPn0572, thus increasing the flux of G-actin required for actin nucleation by CPn0572. F-actin stabilization by cofilin displacement and the nucleation/polymerization activity of CPn0572/Tarp together would therefore be expected to significantly promote formation of highly polymerized and stable F-actin at sites of EB entry. That both functions reside in the CPn0572 protein is unprecedented and may represent an example of the concentration of functional units during genome reduction (Nunes and Gomes, 2014).

In conclusion, our data reveal that the important human pathogen *C. pneumoniae* has evolved an essential effector protein, which (i) re-models the actin cytoskeleton, and (ii) stabilizes F-actin by excluding cofilin. Further studies are required to fully decipher the CPn0572-mediated crosstalk between *C. pneumoniae* and actin dynamics.

## Author contributions

RZ and JHH designed the experiments. RZ conducted the experiments and collected the data. CB performed additional transfection experiments of human cells and did Westernblot analyses of human HEK293 cells expressing GFP-CPn0572 fusion proteins. RZ prepared all figures except Figure S3B which was prepared by CB. RZ and JHH analyzed the data and wrote the manuscript.

### Conflict of interest statement

The authors declare that the research was conducted in the absence of any commercial or financial relationships that could be construed as a potential conflict of interest.
