# Supplementary figures and images for: The Chlamydia pneumoniae Tarp Ortholog CPn0572 Stabilizes Host F-Actin by Displacement of Cofilin
Source: Front Cell Infect Microbiol. 2017 Dec 12;7:511. doi: 10.3389/fcimb.2017.00511 (PMC5770662; doi:10.3389/fcimb.2017.00511)

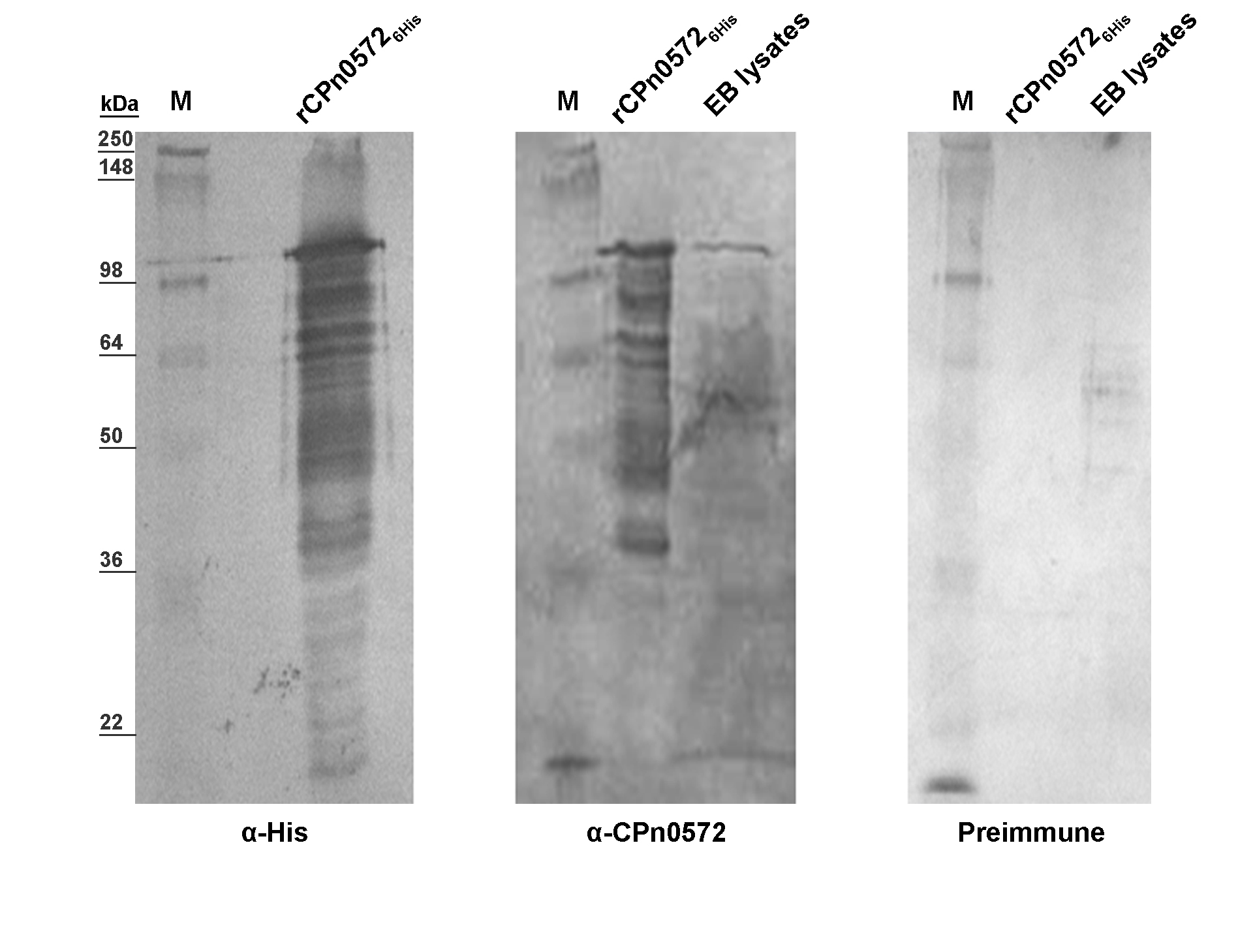

Supplement: Supplementary file 1 [file Image1.JPEG]

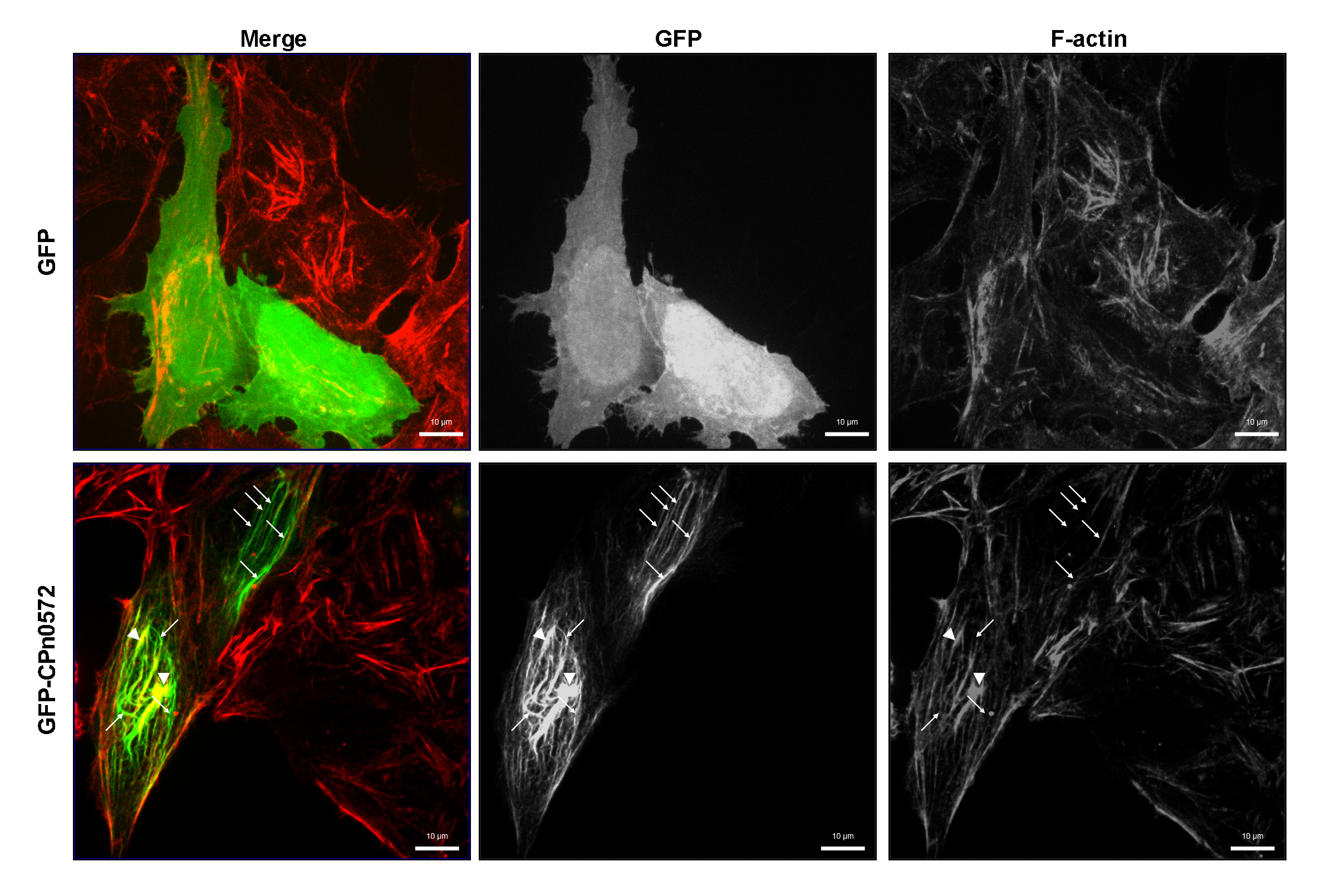

Supplement: Supplementary file 2 [file Image2.JPEG]

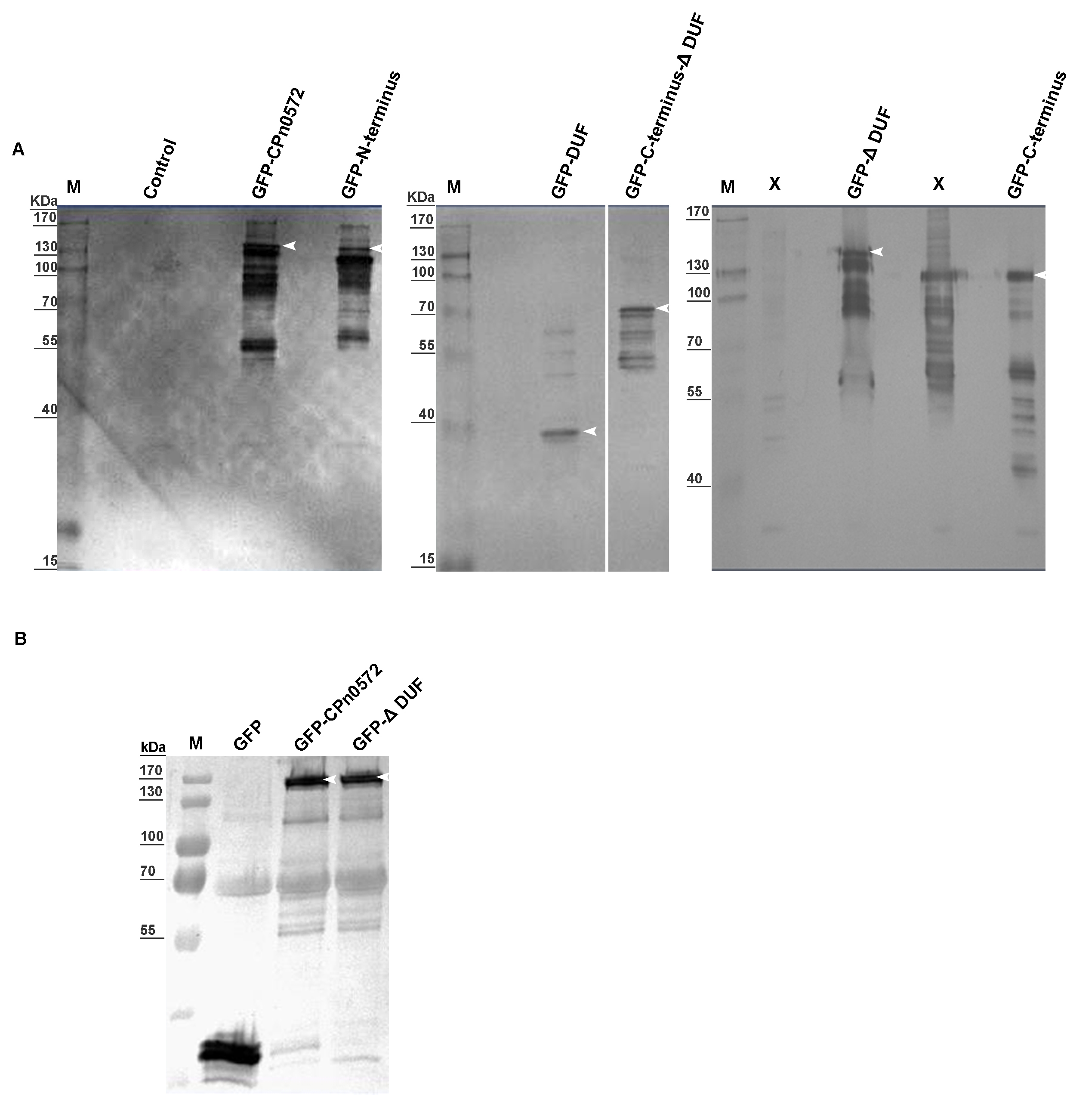

Supplement: Supplementary file 3 [file Image3.JPEG]

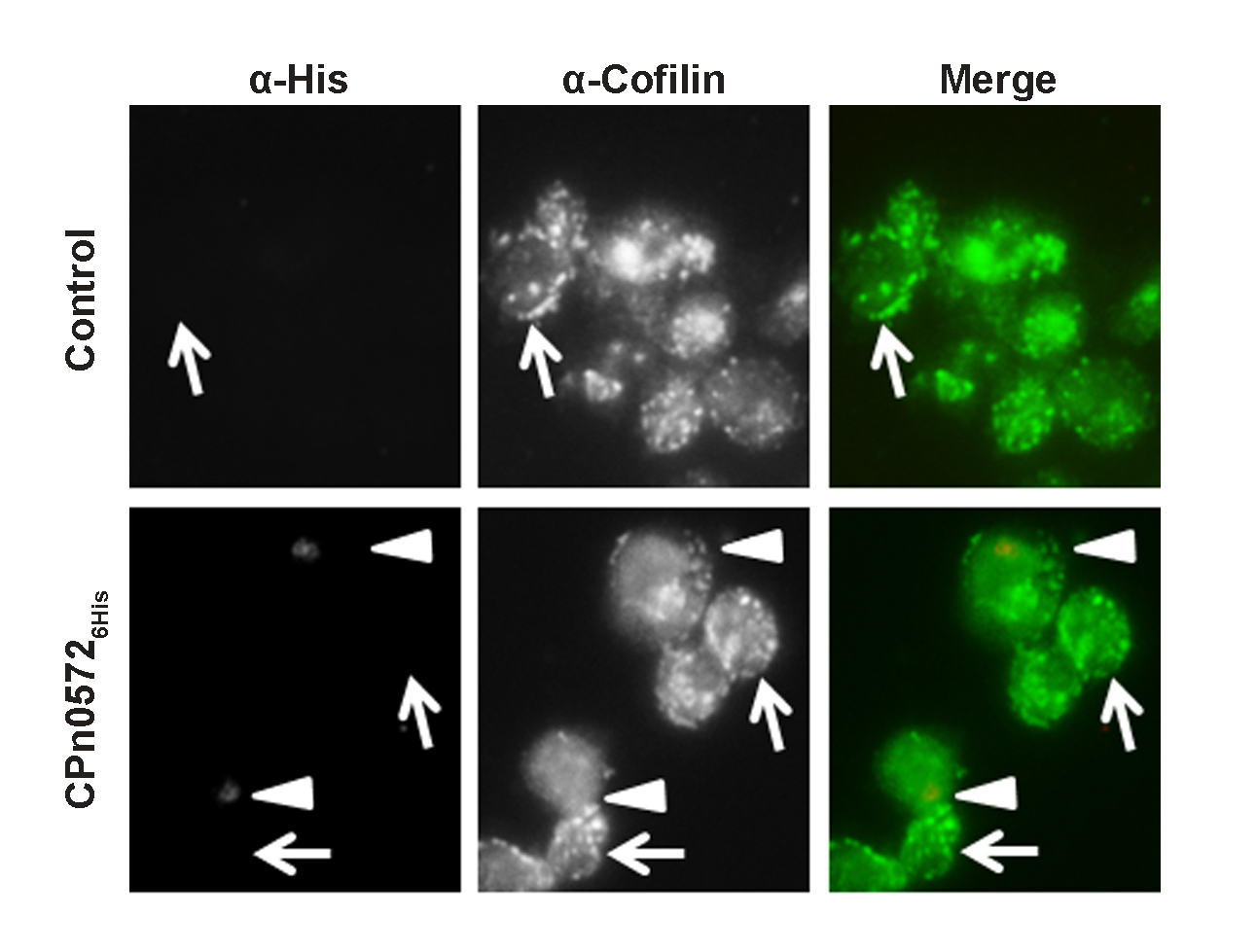

Supplement: Supplementary file 4 [file Image4.JPEG]

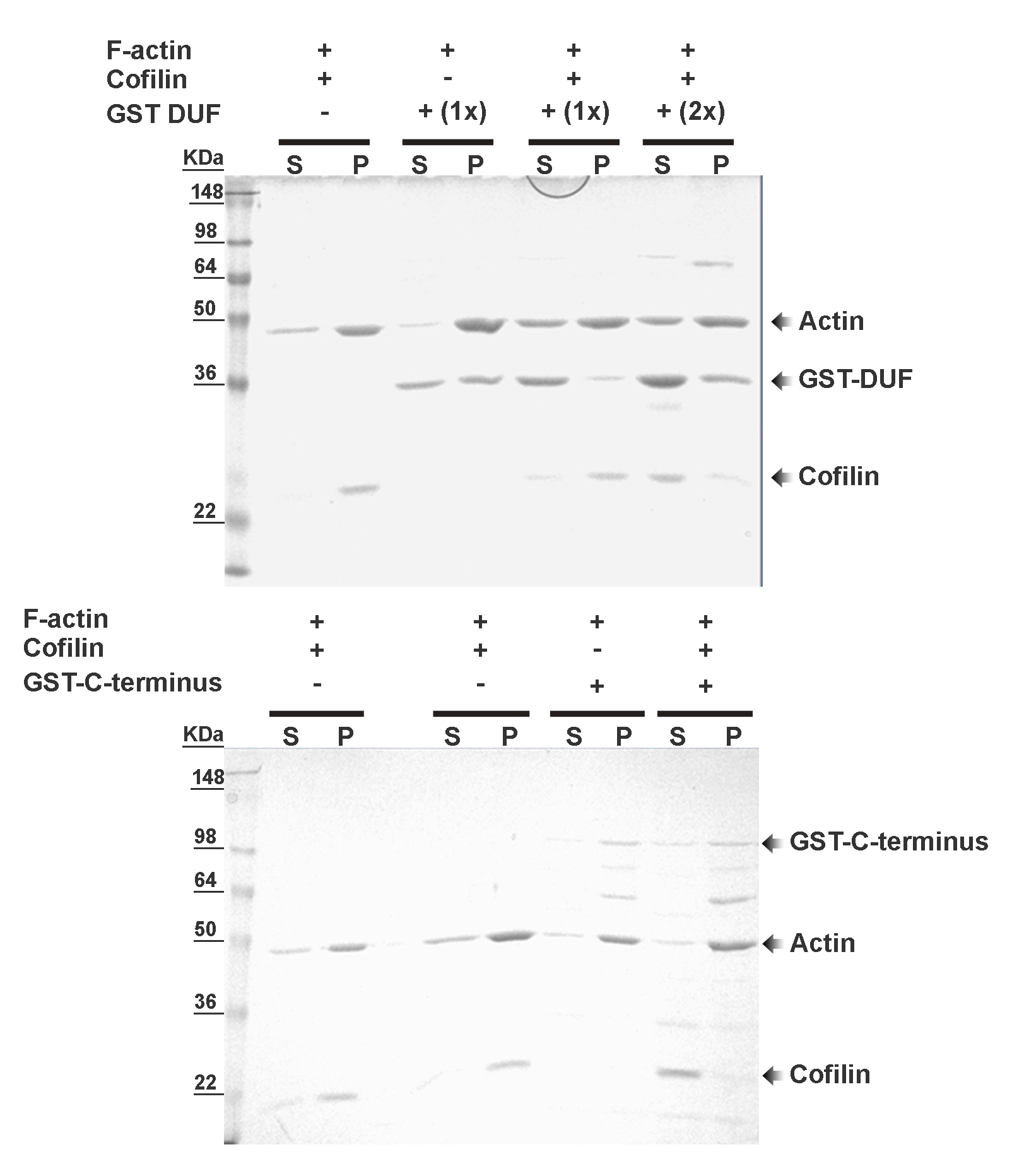

Supplement: Supplementary file 5 [file Image5.JPEG]
