# Supplementary material for: The Chlamydia pneumoniae Tarp Ortholog CPn0572 Stabilizes Host F-Actin by Displacement of Cofilin
Source: Front Cell Infect Microbiol. 2017 Dec 12;7:511. doi: 10.3389/fcimb.2017.00511 (PMC5770662; doi:10.3389/fcimb.2017.00511)
Supplement: Supplementary file 6 [file DataSheet1.DOCX]

**Supplemental Figure legends**

**Figure S1. Full-scale images of the blots presented in Fig.1A**

**Figure S2. GFP-CPn0572 is associated with F-actin in HEp-2 transfected cells.**

HEp-2 cells expressing GFP alone or GFP-CPn0572 were stained with phalloidin to visualize F-actin (red), GFP signals are in green. Note that not all GFP‑CPn0572 filaments (arrows) coincide with actin fibers (arrowheads).

**Figure S3. Expression of CPn0572 and its derivatives in yeast and human cells.**

(A) Crude lysates of yeast cells expressing GFP (control), GFP-CPn0572 and GFP fused to CPn0572 domains were subjected to SDS PAGE. The expression of GFP and the fusion proteins were detected on immunoblots probed with anti-GFP antibodies. Arrowheads indicate the full length bands of the corrosponding protein fragments (X: not relevant). (B) Crude lysates of HEK293T cells expressing GFP (control), GFP-CPn0572 and GFP‑Δ DUF were subjected to SDS PAGE. The expression of GFP and the fusion proteins was detected on immunoblots probed with anti-GFP antibodies. Equal amounts of yeast and human protein extracts were loaded on each lane.

**Figure S4.** **Distribution of cofilin in wild‑type yeast cells (BY4741) expressing CPn0572_6His_.** Cofilin (green) was visualized in wild-type cells carrying the empty vector (control) or the CPn0572_6His_-expressing vector. In wildtype yeast cells cofilin localization is restricted to cortical patches. Yeast cells that expressed CPn0572_6His_ exhibited mostly diffuse staining of cofilin (arrowheads), while non-expressing cells displayed distinct cofilin patches (arrows).

**Figure S5. Full-scale images of the blot presented in Fig. 7.**

**Table1: Plasmid used to generate the constructs required in this work**

| **Plasmid** | **Description** | **Source** |
| --- | --- | --- |
| pAC2 | *E. coli* expression vector. | (Wuppermann et al., 2008) |
| pcDNA3.1/NT-GFP | Mammalian expression vector for creation of N-terminal GFP-containing fusion proteins | Invitrogen |
| pBYE | Mammalian expression vector for creation of N-terminal GFP-containing fusion proteins. A CEN6/ARSH4/URA3 DNA fragment generated by PCR was integrated into the *Dra*III site of pcDNA3.1/NT-GFP (Invitrogen) to allow cloning by homologous recombination in *S. cerevisiae.* | This work |
| p426MET25 | S. *cerevisiae* high-copy-number expression vector | (Mumberg et al., 1994) |
| pUG34 | S. *cerevisiae* expression vector for creation of N-terminal GFP-containing fusion proteins | Ulrich Güldener and Johannes Hegemann, unpublished data |
| p423GAL1 | S. *cerevisiae* high-copy-number expression vector | (Mumberg et al., 1994) |
| pKM36 | *E. coli* expression vector for creation of N-terminal GST-containing fusion proteins | Katja Mölleken and Johannes Hegemann, unpublished data |
| pm-Cherry-ACTIN | Mammalian vector expressing mCherry‑Human actin gene | Scott Grieshaber, University of Florida |

**Table2: plasmids and oligonucleotides used in this work.**

| **Cloned DNA fragments and plasmid** | **Forward and revers primers (5 ’-3 ’) used for the amplification of the cloned DNA fragment** |
| --- | --- |
| *cpn0572* with N-terminal *6x his* in pAC2 | Fwd: GGTGTTTTCACGAGCACTTCACCAACAAGGACCATAGATTATGAGAGGATCG*CATCACCATCACCATCAC*  ATGGCAGCTCCTATCAACCA  Rev: GCCAAGCTTGCCTGCAGGTCGACTCTAGAGGATCCGAATTCTATTTTCTTCGTGGACTTGT |
| *cpn0572* in pBYE | Fwd: GGATGAGCTCTACAAAAGCGGTTCCGGACCGGTGCTAGCG*GCAGCTCCTATCAACCAACC*  Rev: GAATTCCACCACACTGGACTAGTGGATCCGAGCTCGGTACCTATTTTCTTCGTGGACTTGTT |
| *ct456* in pBYE | Fwd: GGATGAGCTCTACAAAAGCGGTTCCGGACCGGTGCTAGCG*ACGAATTCTATATCAGGTGATC*  Rev: GAATTCCACCACACTGGACTAGTGGATCCGAGCTCGGTACTTATCCTACGGTATCAATCAG |
| *cpn0572* lacking *duf* in pBYE | Fwd: GGATGAGCTCTACAAAAGCGGTTCCGGACCGGTGCTAGCG*GCAGCTCCTATCAACCAACC*  Rev: GAATTCCACCACACTGGACTAGTGGATCCGAGCTCGGTACCTATTTTCTTCGTGGACTTGTT |
| *cpn0572* with C-terminal *6x his* in p426MET25 | Fwd: CTATTACCCCCATCCATACTCTAGAACTAGTGGATCCCCCATGGCAGCTCCTATCAACCA  Rev: GGTCGACGGTATCGATAAGCTTGATATCGAATTCCTGCAGCTA*GTGATGGTGATGGTGATG*TTTTCTTCGT GGACTTGTTGG |
| *cpn0572* (nt: 1-1608) with C-terminal *6x his* in p426MET25 | Fwd: CTATTACCCCCATCCATACTCTAGAACTAGTGGATCCCCC*ATGGCAGCTCCTATCAACCA*  Rev: GGTCGACGGTATCGATAAGCTTGATATCGAATTCCTGCAGCTA*GTGATGGTGATGGTGATG*T*GCTTGACC GGATGGATCTG* |
| *cpn0572* (nt: 1432-2268) with C-terminal *6x his* in p426MET25 | Fwd: CTATTACCCCCATCCATACTCTAGAACTAGTGGATCCCCCATGCAAGTCTTACAGAATGTCCGA  Rev: GGTCGACGGTATCGATAAGCTTGATATCGAATTCCTGCAGCTA*GTGATGGTGATGGTGATG*TTTTCTTCGT GGACTTGTTGG |
| *cpn0572* (nt: 1432-1608) with C-terminal *6x his* in p426MET25 | Fwd: CTATTACCCCCATCCATACTCTAGAACTAGTGGATCCCCCATGCAAGTCTTACAGAATGTCCGA  Rev: GGTCGACGGTATCGATAAGCTTGATATCGAATTCCTGCAGCTA*GTGATGGTGATGGTGATG*TGCTTGACC  GGATGGATCTG |
| *cpn0572* (nt: 1609-2268) with C-terminal *6x his* in p426MET25 | Fwd: CTATTACCCCCATCCATACTCTAGAACTAGTGGATCCCCCATGACCGGAGGAGTCACTGA  Rev: GGTCGACGGTATCGATAAGCTTGATATCGAATTCCTGCAGCTA*GTGATGGTGATGGTGATG*TTTTCTTCGT GGACTTGTTGG |
| 🞻*cpn0572* (nt: 1-1431 + 1609-2268) with C-terminal *6x his* in p426MET25 | Fragment 1  Fwd: CTATTACCCCCATCCATACTCTAGAACTAGTGGATCCCCCATGGCAGCTCCTATCAACCA  Rev: TATTACGGATATGACCGCCGCCTTCAGTGACTCCTCCGGTGTTCAAGGACGCATTCGAAAT  fragment 2:  Fwd: ACCGGAGGAGTCACTGAAGG  Rev: GGTCGACGGTATCGATAAGCTTGATATCGAATTCCTGCAGCTA*GTGATGGTGATGGTGATG*TTTTCTTCGT GGACTTGTTGG |
| *cpn0572* in pUG34 | Fwd: TATGGATGAATTGTACAAATCTAGAACTAGTGGATCCCCCATGGCAGCTCCTATCAACCA  Rev: CGACGGTATCGATAAGCTTGATATCGAATTCCTGCAGCCCCTATTTTCTTCGTGGACTTGT |
| *cpn0572* (nt: 1-1608) in pUG34 | Fwd: TATGGATGAATTGTACAAATCTAGAACTAGTGGATCCCCCATGGCAGCTCCTATCAACCA  Rev: CGACGGTATCGATAAGCTTGATATCGAATTCCTGCAGCCCCTATGCTTGACCGGATGGATCTG |
| *cpn0572* (nt: 1432-2268) in pUG34 | Fwd: TATGGATGAATTGTACAAATCTAGAACTAGTGGATCCCCCCAAGTCTTACAGAATGTCCGA  Rev: CGACGGTATCGATAAGCTTGATATCGAATTCCTGCAGCCCCTATTTTCTTCGTGGACTTGT |
| *cpn0572* (nt: 1432-1608) in pUG34 | Fwd: TATGGATGAATTGTACAAATCTAGAACTAGTGGATCCCCCCAAGTCTTACAGAATGTCCGA  Rev: CGACGGTATCGATAAGCTTGATATCGAATTCCTGCAGCCCCTATGCTTGACCGGATGGATCTG |
| *cpn0572* (nt: 1609-2268) in pUG34 | Fwd: TATGGATGAATTGTACAAATCTAGAACTAGTGGATCCCCCACCGGAGGAGTCACTGAAGG  Rev: CGACGGTATCGATAAGCTTGATATCGAATTCCTGCAGCCCCTATTTTCTTCGTGGACTTGT |
| *cCpn0572* in pKM36 | Fwd: TCCTCCAAAATCGGATCTGATCGAAGGTCGTGGGATCCCCCTGGAAGTTCTGTTCCAGGGGCCCCTG  GGATCCATGGCAGCTCCTATCAACCAA  Rev: ACGCGCGAGGCAGATCGTCAGTCAGTCACGATGAATTCCCCTATTTTCTTCGTGGACTTGTT |
| *cpn0572* (nt: 1432-2268) in pKM36 | Fwd: TCCTCCAAAATCGGATCTGATCGAAGGTCGTGGGATCCCCCTGGAAGTTCTGTTCCAGGGGCCCCTG  GGATCCATGCAAGTCTTACAGAATGTCCGAC  Rev: ACGCGCGAGGCAGATCGTCAGTCAGTCACGATGAATTCCCCTATTTTCTTCGTGGACTTGTT |
| *cpn0572* (nt: 1432-1608) in pKM36 | Fwd: TCCTCCAAAATCGGATCTGATCGAAGGTCGTGGGATCCCCCTGGAAGTTCTGTTCCAGGGGCCCCTG  GGATCCATGCAAGTCTTACAGAATGTCCGAC  Rev: ACGCGCGAGGCAGATCGTCAGTCAGTCACGATGAATTCCCCTATGCTTGACCGGATGGATCTG |
| *cpn0572* (nt: 1027-1608) in pKM36 | Fwd: TCCTCCAAAATCGGATCTGATCGAAGGTCGTGGGATCCCCCTGGAAGTTCTGTTCCAGGGGCCCCTG  GGATCCTCCTTTATAAATGAAACTCCAG  Rev: ACGCGCGAGGCAGATCGTCAGTCAGTCACGATGAATTCCCCTATGCTTGACCGGATGGATCTG |

Fwd: forward primer.

Rev: reverse primer.

Priming nucleotide homology to the target DNA is underlined.

Nucleotides coding for *6x his* are in italic*.*

🞻 The 2 fragments in this construct were integrated into the plasmid via triple homologous recombination in yeast.
